# Quasi van der Waals Epitaxy of Single Crystalline GaN on Amorphous SiO_2_/Si(100) for Monolithic Optoelectronic Integration

**DOI:** 10.1002/advs.202305576

**Published:** 2024-03-22

**Authors:** Dongdong Liang, Bei Jiang, Zhetong Liu, Zhaolong Chen, Yaqi Gao, Shenyuan Yang, Rui He, Lulu Wang, Junxue Ran, Junxi Wang, Peng Gao, Jinmin Li, Zhongfan Liu, Jingyu Sun, Tongbo Wei

**Affiliations:** ^1^ Research and Development Center for Semiconductor Lighting Technology Institute of Semiconductors Chinese Academy of Sciences Beijing 100083 P. R. China; ^2^ Center of Materials Science and Optoelectronics Engineering University of Chinese Academy of Sciences Beijing 100049 P. R. China; ^3^ Center for Nanochemistry (CNC) Beijing Science and Engineering Center for Nanocarbons Beijing National Laboratory for Molecular Sciences College of Chemistry and Molecular Engineering Peking University Beijing 100871 P. R. China; ^4^ Beijing Graphene Institute (BGI) Beijing 100095 P. R. China; ^5^ Electron Microscopy Laboratory and International Center for Quantum Materials School of Physics Peking University Beijing 100871 P. R. China; ^6^ State Key Laboratory of Superlattices and Microstructures Institute of Semiconductors Chinese Academy of Sciences Beijing 100083 P. R. China; ^7^ College of Energy Soochow Institute for Energy and Materials InnovationS (SIEMIS) Jiangsu Provincial Key Laboratory for Advanced Carbon Materials and Wearable Energy Technologies Soochow University Suzhou 215006 P. R. China

**Keywords:** GaN, graphene, monolithic integration, quasi van der waals epitaxy, Si(100)

## Abstract

The realization of high quality (0001) GaN on Si(100) is paramount importance for the monolithic integration of Si‐based integrated circuits and GaN‐enabled optoelectronic devices. Nevertheless, thorny issues including large thermal mismatch and distinct crystal symmetries typically bring about uncontrollable polycrystalline GaN formation with considerable surface roughness on standard Si(100). Here a breakthrough of high‐quality single‐crystalline GaN film on polycrystalline SiO_2_/Si(100) is presented by quasi van der Waals epitaxy and fabricate the monolithically integrated photonic chips. The in‐plane orientation of epilayer is aligned throughout a slip and rotation of high density AlN nuclei due to weak interfacial forces, while the out‐of‐plane orientation of GaN can be guided by multi‐step growth on transfer‐free graphene. For the first time, the monolithic integration of light‐emitting diode (LED) and photodetector (PD) devices are accomplished on CMOS‐compatible SiO_2_/Si(100). Remarkably, the self‐powered PD affords a rapid response below 250 µs under adjacent LED radiation, demonstrating the responsivity and detectivity of 2.01 × 10^5^ A/W and 4.64 × 10^13^ Jones, respectively. This work breaks a bottleneck of synthesizing large area single‐crystal GaN on Si(100), which is anticipated to motivate the disruptive developments in Si‐integrated optoelectronic devices.

## Graphical abstract

The work breaks a bottleneck of synthesizing high quality single‐crystal GaN on amorphous SiO_2_/Si(100) and demonstrates the monolithically integrated photonic chips on CMOS‐compatible substrate for the first time. The self‐powered PD affords a rapid response below 250 µs under adjacent LED radiation, demonstrating the high responsivity and detectivity of 2.01 × 10^5^ A W^−1^ and 4.64 × 10^13^ Jones.

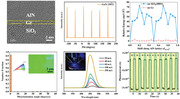

## 1 Introduction

Because of their outstanding properties, GaN‐based semiconductors have attracted significant attention in the fields of optoelectronic, high‐frequency, and high‐power applications.^[^ ^1^ ^−^ ^3^ ^]^ In this context, heterogeneous integration of GaN‐based devices with Si‐based complementary metal‐oxide‐semiconductor (CMOS) holds great promise for enhancing system speed and reducing power consumption.^[^ ^4^, ^5^ ^]^ The integration of the standard Si(100) CMOS with GaN‐based devices would undoubtedly promote advanced semiconductor technology, with the potential to overcome the limitation of Moore's law. Recently, great efforts have been made to integrate GaN epilayers on Si(100) substrate.^[^ ^6^, ^7^ ^]^ In comparison with the wafer bonding techniques, which entail complex processes and high costs, direct hetero‐epitaxy of (0001) GaN on Si(100) is preferrable, due to its compatible with Si integrated circuits (ICs).^[^ ^8^, ^9^ ^]^

To date, unlike the mature epitaxial technology on Si(111) substrate, the epitaxial growth of GaN(0001) on Si(100) platform has still faced key obstacles owing to high lattice/thermal mismatch and distinct crystal symmetry. As a consequence, GaN epilayer exhibits a pronounced susceptibility to crackings and a high density of dislocations.^[^ ^10^, ^11^ ^]^ Since the Si(100) substrate possesses a tetragonal symmetry with a 2 × 1 surface reconstruction, polycrystalline GaN with two misoriented domains could be easily formed, showing a rough surface and poor crystallinity. To exert the control over the domain orientation of GaN, patterned Si(100) with the micrometer‐sized or miscut Si(100) have been explored, but faces with severely anisotropic performance and unresolved reliability issues.^[^ ^12^, ^13^ ^]^ The presence of a native oxide layer on the Si(100) surface further complicates the situation by hindering conventional epitaxial interactions, resulting in random orientations and rough morphologies of as‐grown GaN. Despite fruitful achievements in forming hexagonal GaN films on Si(111) substrate, the growth of high‐quality single‐crystalline GaN on Si(100) remains rather elusive.^[^ ^14^ ^]^

To this end, an emerging quasi‐van der Waals (qvdW) epitaxy or remote epitaxy (RE) route based upon 2D architectures has been proposed to allow fine heteroepitaxial growth of group‐III nitrides.^[^ ^15^, ^16^, ^17^, ^18^, ^19^ ^]^ The 2D materials harnessing hexagonal symmetry might not only screen the misorientation effect of underlying substrates such as SiO_2_/Si(100), but also facilitates the formation of wurtzite nitrides.^[^ ^20^, ^21^, ^22^ ^]^ Depositing nearly single‐crystalline GaN films on amorphous substrates by inserting a transferred graphene (Gr) buffer layer was realized, with the aid of AlGaN nanorods or ZnO nanowalls.^[^ ^20^, ^23^, ^24^, ^25^ ^]^ Nevertheless, how to precisely control the out‐of‐plane and in‐plane orientations of high‐quality (0001) GaN via Gr on the amorphous supports is ambiguous. Note further that all these employed Gr buffer layers are grown on metals and transferred onto target substrate by a complicated wet‐etching process, which inevitably generates high‐cost aspects and introduces foreign contaminants.

In this work, we realize direct qvdW growth of high quality single‐crystalline GaN film and present the monolithic integration of photodetector (PD) and light‐emitting diode (LED) on SiO_2_/Si(100), rendering markedly improved GaN quality as compared to previous reports.^[^ ^20^, ^23^, ^26^, ^27^, ^28^, ^29^, ^30^ ^]^ Uniform and continuous Gr interlayer is directly grown on SiO_2_/Si(100) wafer without a metal catalyst, ensuring the compatibility with metal–organic chemical vaper deposition (MOCVD) process. The out‐of‐plane orientation of GaN is guided by Gr and multi‐step growth, while the in‐plane orientation is aligned through the slip and rotation process of AlN nucleation islands due to the weakened qvdW interface interaction. The monolithic integration of prototype devices on CMOS‐compatible SiO_2_/Si(100) is demonstrated. Under adjacent LED radiation, the photo‐to‐dark‐current ratio (PDCR) value of the self‐powered PD is 12, and the response time is about 30 ms. Our results hold great potential in the marriage of GaN‐based devices with Si(100)‐based ICs, which is anticipated to ultimately motivate conspicuous advances in optoelectronic integrated devices.

## 2 Results

**Figure 1:**
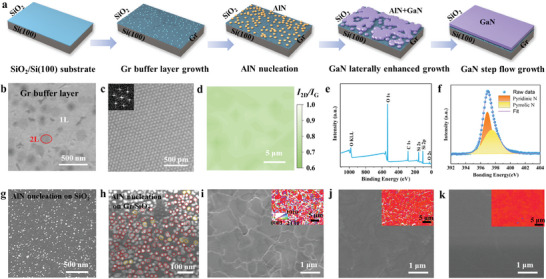
The epitaxy of continuous GaN film on SiO_2_/Si(100) substrate with the assistance of a directly grown Gr interlayer. a) Schematic diagram of the key processes of epitaxy of GaN film on SiO_2_/Si(100) substrate. b) SEM image of the directly grown Gr on SiO_2_/Si(100), indicating the substrate is fully covered by Gr. c) Atomically resolved TEM image of directly grown Gr after transferring onto TEM copper grid. The inset shows the corresponding FFT pattern of Gr. d) Raman mapping of I_D_/I_G_ of Gr film in a 20 × 20 µm^2^ region confirming the uniformity of Gr. e) The typical XPS spectrum of directly grown Gr. f) XPS spectrum of N_1s_ of AlN/Gr/SiO_2_/Si(100) after an annealing process under NH_3_ atmosphere. g,h) SEM images of AlN nucleation on SiO_2_/Si(100) without and with the N‐doped Gr interlayer (the orientations of AlN islands labeled by different colors). i) SEM image of the initial grown GaN crystal domains on AlN/Gr/SiO_2_/Si(100). j) Coalescence of GaN domains with enhanced lateral growth rate. k) SEM image of the smooth surface of GaN film grown on AlN/Gr/SiO_2_/Si(100) after the step‐flow growth process. Insets in i–k: Corresponding out‐of‐plane EBSD maps of GaN films.

**Figure** **1**a schematically depicts the key processes involved in the growth of single‐crystalline GaN film on Gr‐coated substrate. The fabrication process begins with the direct growth of a continuous Gr film on SiO_2_/Si(100) substrate by metal‐free chemical vapor deposition (CVD), as revealed by scanning electron microscopy (SEM) image (Figure 1b). The layer thickness features (mainly 1–2 layers) of Gr are further confirmed by the transmission electron microscopy (TEM) characterization (Figures 1c; S1a, Supporting Information). Although its imaging principle causes deviations in lattice spacing compared to actual graphene, the identification of graphene can be confirmed through the observation of FFT patterns in the inset of Figure 1c. The Raman mapping results (Figure 1d) with typical Raman spectrum (Figure S1b, Supporting Information) suggest the uniform coverage of Gr at a large scale, displaying characteristic peaks at D‐ (1349 cm^−1^), G‐ (1590 cm^−1^), and 2D‐ (2685 cm^−1^) bands. Note that the *I*_2D_/*I*_G_ ratio does not reach roughly 2 (typical monolayer Gr feature), which is possibly attributed to the non‐uniformity of the layer numbers of directly grown Gr film (mainly 1–2 layers with the occurrence of 3–5 layers). It is a general consensus that the precise layer number control of the direct growth of Gr over SiO_2_/Si substrate is highly unlikely. X‐ray photoelectron spectroscopy (XPS) analysis shows no detectable metal elements in the as‐grown Gr film that may hinder subsequent nitride growth (Figure 1e). As a non‐polar 2D sheet without dangling bonds, pristine Gr would restricts the nucleation of adatoms on its passive surface. An in situ annealing process is accordingly developed to enhance the nucleation of nitrides on the Gr surface, in which N dangling bonds are introduced into Gr by NH_3_‐treatment at 1100 °C in a growth chamber (Figure 1f). In detail, the component peaks in N_1s_ XPS spectrum at 396.9 and 397.5 eV are ascribed to the nitrogen‐doped Gr with pyridinic and pyrrolic C–N bonding.^[^ ^31^, ^32^ ^]^ For comparison, AlN is also simultaneously grown on SiO_2_/Si(100) substrate without Gr interlayer in the same trial by MOCVD. As shown in Figures 1g,h, high nuclei density of AlN islands grown at 1200 °C can be observed on Gr‐covered SiO_2_/Si(100) with regular hexagonal shape. The size of islands varies in the range of ≈25–35 nm, which almost keeps identical in‐plane orientation. High density of nucleation sites on N‐doped Gr and high growth temperature ensure AlN islands to align with the approximately preferred orientation. In contrast, due to the lack of epitaxial relationship between AlN and amorphous SiO_2_, there are only randomly oriented nuclei with sparse distribution on bare SiO_2_/Si(100). The mechanism of enhanced nucleation and well‐aligned orientation of AlN nuclei on N‐doped Gr will be discussed later in detail.

Benefiting from quite low surface energy in MOCVD growth, these AlN nuclei on Gr prefer c‐axis orientation and lay a good foundation for the subsequent merge of GaN.^[^ ^30^, ^33^ ^]^ Single‐crystalline GaN film is further realized on the AlN nuclei throughout multi‐step growth procedures. First, the GaN experiences a surface coarsening growth stage, where quasi‐3D island formation gradually dominates (Stranski–Krastanov growth mode) at a high temperature and low V/III ratio, growing into a continuous GaN layer with a roughened surface (Figure 1i). Partial strain in the GaN epilayer can be released by the formation of dislocations at this stage. As for the following recovery growth stage, most of the dislocations and defects in GaN film are eliminated with a high V/III ratio under a higher temperature and lower pressure (Figure 1j). In detail, some threading dislocations are bent to form a dipole half‐loop due to their image force. Moreover, if two dislocation lines have the opposite Burger vectors, they would be annihilated with each other.^[^ ^33^ ^]^ Finally, in the 2D growth stage, GaN is deposited at high temperature and low pressure to ensure a completely smooth film surface (Figure 1k). In addition, the electron backscatter diffraction (EBSD) mapping of thus‐obtained GaN films demonstrates a whole evolution of c‐axis preferred orientation with the increase of GaN thickness, revealing its single‐crystalline feature after the step‐flow growth (inset in Figures 1i–k). With respect to the entire growth process, the c‐axis dominant orientation originated from on AlN seeding on Gr is gradually strengthened and the indiscriminate nuclei can also be engulfed by the dominant one in the multi‐step growth process. Leung et al. also noted that with increasing epilayer thickness, nuclei with oblique growth axes were selectively displaced by neighboring nuclei showing superior on‐axis alignment.^[^ ^9^ ^]^ However, the GaN grown on SiO_2_/Si(100) without Gr shows a lumpy polycrystalline morphology (Figure S2, Supporting Information). It is noted that the in‐plane and out‐of‐plane orientations of these GaN films are random in this case, due to the lack of epitaxial relationship between the substrate and epitaxial layer (Figures S2 and S3, Supporting Information). Therefore, one can conclude that using transfer‐free Gr as the interlayer and high temperature AlN as the seeding layer, continuous and smooth GaN film can be obtained on the amorphous SiO_2_/Si(100), accompanied by the enhanced lateral growth rate.

**Figure 2:**
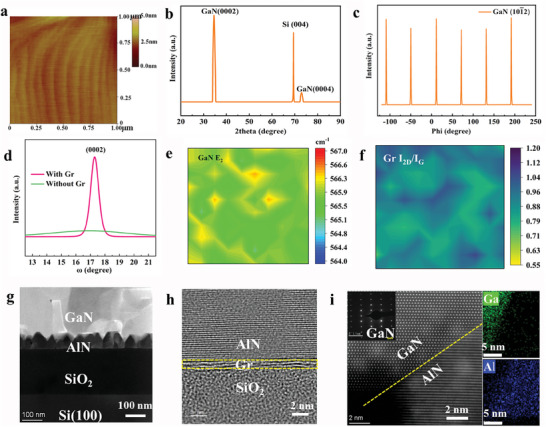
Characterization of single‐crystalline GaN film grown on SiO_2_/Si(100) substrate with the assistance of Gr interlayer. a) AFM image of the as‐grown GaN film on SiO_2_/Si(100) substrate, showing a step‐flow surface morphology with an RMS of 0.185 nm. b) XRD omega/2theta scan curve of GaN. Peaks correspond to (0002) GaN, (004) Si and (0004) GaN, respectively. c) XRD ϕ‐scan of (10$\bar{1}$2) GaN planes, confirming the single crystalline feature of as‐grown GaN film on Gr/SiO_2_/Si(100). d) The X‐ray rocking curve of (0002) GaN peak of as‐grown GaN films with and without Gr interlayers, and the FWHM with Gr is 0.46°. e,f) Raman mapping of GaN E_2_ peak (e) and *I*_2D_/*I*_G_ of Gr (f) after the GaN growth process. g) STEM‐HAADF image of the interface (layers from bottom to top are crystalline Si, amorphous SiO_2_, crystalline AlN, and crystalline GaN, respectively). h) The HRTEM image of AlN/Gr/SiO_2_ interface, confirming the existence of Gr structure. i) STEM‐HAADF image of GaN/AlN interface and the corresponding EDS mapping of Ga (green) and Al (blue) elements.

To evaluate the degree of single crystallinity, the obtained GaN films on Gr/SiO_2_/Si(100) are characterized by atomic force microscopy (AFM), X‐ray diffraction (XRD), TEM, and Raman spectroscopy. **Figures** **2**a and S4 (Supporting Information) shows the AFM image of the GaN surface morphology, revealing the root mean square (RMS) roughness of only 0.185 nm in a scanned area of 1 ×1 µm^2^. Clear atomic step terraces also indicate the atomically flat surface of obtained GaN film. The growth orientation of GaN epilayer is characterized by XRD. Figure 2b shows the diffraction peaks of (0002) and (0004) of wurtzite GaN, indicating the uniform out‐of‐plane oriented hexagonal structure of flat GaN film. Furthermore, GaN (10$\bar{1}$2)‐plane ϕ scan is applied to verify the in‐plane orientation of the GaN epilayer, which only shows one set of pronounced peaks with intervals of 60° (Figure 2c). This confirms the uniform in‐plane alignment, ensuring the single‐crystalline GaN film on Gr/SiO_2_/Si(100). The X‐ray rocking curve full width at the half maximum (FWHM) of the (0002) peak of GaN film with Gr interlayer is 0.46° as shown in Figure 2d, further confirming high‐quality GaN film on Gr/SiO_2_/Si(100). In contrast, without the Gr interlayer, GaN (10$\bar{1}$2)‐plane ϕ scan shows some miscellaneous peaks, implying the GaN film grown directly on bare SiO_2_/Si(100) is polycrystalline (Figure S5, Supporting Information). The summary of the recent process of GaN epilayers on amorphous substrates is listed in Table S1 (Supporting Information). These results prove the potential of the 2D Gr to enable the epitaxy of the crystalline III‐nitride films on amorphous SiO_2_/Si(100), which breaks the limitation of conventional epitaxy rules.^[^ ^34^, ^–36^ ^]^ Besides, the as‐grown GaN film on Gr/SiO_2_/Si(100) can be easily transferred onto arbitrary target substrates by a facile mechanical lift‐off, using thermally release tape as supporting layer.^[^ ^37^, ^38^, ^39^ ^]^ Wafer‐scale exfoliated GaN membrane will be discussed in another work.

We further investigate the strain relaxation of GaN film grown on SiO_2_/Si(100) with Gr interlayer. Figure S6 (Supporting Information) indicates that the GaN film grown on Gr‐coated substrate undergoes slight tensile strain, showing a smaller *E*_2_ frequency (565.9 cm^−1^) compared to bulk (567.0 cm^−1^). The frequency deviation, Δ*ω*, compared to strain‐free intrinsic GaN can be used to estimate the biaxial stress σ_xx,_ according to σ_xx_ = Δω/*K*, where *K* is the biaxial stress factor (2.56 cm^−1^ GPa^−1^ for GaN).^[^ ^40^ ^]^ The biaxial stress of GaN grown on Gr/SiO_2_/Si(100) is estimated to be 0.43 GPa, less than the conventional epitaxial GaN film on sapphire.^[^ ^40^ ^]^ Moreover, Raman mapping shows that the stress of GaN film is further relaxed at grain boundaries in Figure 2e. The reduced stress of GaN epilayer can be attributed to the spontaneous relaxation of misfit strain because of the slippery Gr interface.^[^ ^41^ ^]^ In addition, the Gr interlayer is still relatively uniform upon the GaN growth, as evidenced by the Raman *I*_2D_/*I*_G_ mapping (Figure 2f), indicating that the GaN film is indeed grown above the Gr interlayer.^[^ ^42^ ^]^ TEM is used to examine the epitaxy relationship between epilayer and Gr/SiO_2_/Si(100) substrate. A scanning transmission electron microscopy (STEM) high‐angle annular dark‐field (HAADF) image in Figure 2g shows the sample stack of crystalline Si(100), amorphous SiO_2_, crystalline AlN and crystalline GaN. The corresponding energy dispersive spectroscopy (EDS) mapping confirms the elemental distribution of the GaN epilayer (Figure S7, Supporting Information). In contrast, the cross‐section image of GaN without Gr interlayer shows individual polycrystalline domains. A high‐resolution TEM (HRTEM) image clearly shows the atomic planes of the bilayer Gr and AlN (Figure 2h, Figure S8, Supporting Information), forming a perfectly sharp and uniform interface.^[^ ^43^ ^]^ The Gr interlayer remains its two‐atom‐thickness after the epitaxy process. The interface between GaN and AlN is revealed by cross‐sectional STEM‐HAADF and EDS mapping in Figure 2i, showing a good alignment. Notably, the selected area electron diffraction pattern (SAED) also confirms the epitaxial growth direction of GaN is along (0002) (*c*‐axis‐orientation) (inset in Figure 2i).

**Figure 3:**
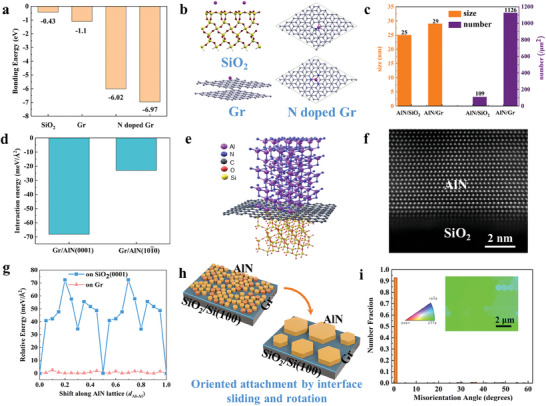
The growth mechanism of single crystalline GaN on SiO_2_/Si(100) with the assistance of Gr. a) The adsorption energies of Al atoms on the SiO_2_ substrate, pristine Gr, and N‐doped Gr after NH_3_ annealing, suggesting the nucleation of AlN is greatly enhanced by N dopant. b) Atomic structures of Al absorbed on different substrates. c) Density and size distribution analysis of AlN nucleation on SiO_2_/Si(100) with and without N doped Gr. d) The interaction energies between Gr and AlN with typical lattice facets, indicating the out‐of‐plane direction of AlN crystal can be controlled by Gr. e) Schematic of stable structure of wurtzite AlN on Gr‐covered polycrystalline SiO_2._ f) Cross‐sectional STEM‐HAADF image of AlN/Gr/SiO_2_ interface, revealing the uniform out‐of‐plane orientation of AlN. C element is too light to be observed compared with Al as the intrinsic contrast is correlated with the atomic number Z. g) Energy barriers for the interface sliding of (0001) AlN film on Gr and bare SiO_2_. h) Schematic of the oriented attachment driving alignment of AlN in‐plane orientation. i) The EBSD image and the corresponding misorientation angles statistics of as‐grown GaN films on SiO_2_/Si(100) after the whole growth process in an area of 8 × 10 µm^2^ showing the predominant in‐plane orientation of the GaN film.

The N dopant in Gr introduced by in‐situ NH_3_ annealing process can greatly enhance the AlN nucleation density. To verify the effects of the N defects on the AlN nucleation, we perform density functional theory (DFT) calculations to compare Al adsorption energies on bare SiO_2_, pristine Gr and N‐defected Gr, as shown in **Figures** **3**a,b. We adopt the stable reconstruction of (0001) SiO_2_ surface as a prototype surface of SiO_2_ substrate. The adsorption energies of Al atom on bare (0001) SiO_2_ substrate and pristine Gr are around −0.43 and −1.1 eV, respectively. This indicates that Al atom is quite difficult to adsorb on the bare SiO_2_ surface. However, Al atom is easily captured by N defects in Gr. We obtain two stable adsorption configurations of Al atom on pyrrolic N (N‐sp^3^) defect, with strong binding energies of −6.97 and −6.02 eV, respectively. Upon the adsorption of a second Al atom, the average binding energies are between −3.83 and −5.33 eV. Besides, the Al atom adsorption energy on pyridinic N (N‐sp^2^) defects can reach −5.50 eV. These results suggest that the enhanced adsorption of Al atoms at N defects can facilitate the nucleation of AlN seeds by the formation of Al‐N‐C bonds (Figure 3b). As a result, the nuclei of AlN on N‐doped Gr show much higher density than that on bare SiO_2_/Si(100) (Figure 3c).

Then, the out‐of‐plane orientation of AlN is investigated by calculating the interaction energy between Gr and typical lattice facets of AlN based on DFT method, and plan‐view HRTEM analysis. We find the (0001) AlN facet shows the strongest interface interaction with Gr (Figure 3d), which is about three times larger than the (10$\bar{1}$0) AlN facet. Thus, the out‐of‐plane direction of AlN crystal can be controlled by Gr, as schematically illustrated in Figure 3e. The STEM‐HAADF image of AlN/Gr/SiO_2_ interface also confirms that AlN is grown along [0001] direction with uniform out‐of‐plane orientation (Figure 3f). Finally, we propose that the in‐plane orientation of each AlN crystal is aligned by oriented attachment under high temperature on slippery Gr surface.^[^ ^44^, ^45^ ^]^ The weakened interface between epitaxial films and substrate due to Gr interlayer may provide a new pathway for in‐plane alignment through such a weak epilayer‐Gr interface. If the energy barrier for rotation or slip of the AlN crystal at the interface is weaker than that required to introduce a grain boundary and can be overcome at high temperature, the AlN crystal would be aligned to form a single crystalline through seamless stitch, although Gr interlayer is polycrystalline on a large scale. We first model two simple (10$\bar{1}$0) AlN boundaries: one is due to the lateral displacement of two (10$\bar{1}$0) AlN facets, and the other is due to a large interface distance. The corresponding boundary energies are calculated to be as large as 166.0 and 268.3 meV/Å^2^, respectively (Figure S9, Supporting Information). The two simple models give a rough estimation of the grain bound energy for AlN films. DFT results confirm our conclusion that interface displacement of AlN slab from one atomic site to the others on Gr surface is much lower than that on bare SiO_2_/Si(100) substrate and is much smaller than that required for the introduction of a grain boundary. As shown in Figure 3g, the calculated maximum energy barrier for the interface slipping of (0001) AlN on Gr is only 2.5 meV Å^−2^ (Figure S10, Supporting Information). We also calculate the energy barrier of rotation by rotating an (0001) AlN nanorod on Gr with respect to its center. The rotational angle between AlN and Gr lattice vectors varies from 0° to 60° (Figure S11, Supporting Information). The maximum rotation energy barrier on Gr is calculated to be 16.0 meV Å^−2^, also much smaller than the energy required for introducing a grain boundary. Due to the small energy barriers of slip and rotation of (0001) AlN on Gr, in‐plane orientation of small AlN nuclei can be easily aligned and incorporated by bigger islands through a slip and rotation process under high temperature as schematically shown in Figure 3h, which is typically called oriented attachment.^[^ ^44^, ^45^ ^]^ Then, large islands will coalesce with the increase of lateral growth rate. In contrast, the slip and rotation of AlN on SiO_2_ substrate are very difficult. As shown in Figure 3g, the energy barrier for the slip of (0001) AlN on bare (0001) SiO_2_ reconstructed surface is 72.4 meV Å^−2^. Therefore, AlN nuclei hardly align to each other on SiO_2_ substrate. The orientation of subsequent GaN would follow the crystal orientation of AlN islands, and thus single‐crystalline GaN film can be obtained on Gr covered SiO_2_/Si(100) substrate targeting photoelectric integration (Figure 3i). Moreover, the multi‐step procedure for GaN growth is also important for reducing the dislocation density by “dislocation interaction” and obtaining smooth surface as discussed above.

**Figure 4:**
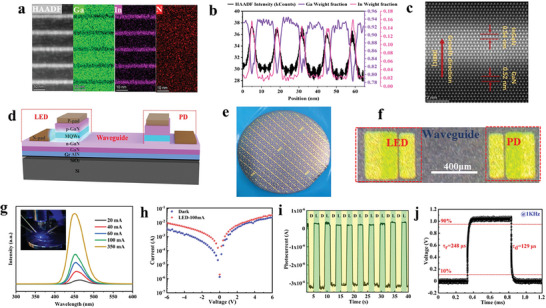
Monolithic integration of GaN‐based LED and PD on Gr‐covered SiO_2_/Si(100). a) Cross‐sectional STEM‐HAADF image of blue‐LED MQWs. b) Line profile of the EDS intensity at the same area. c) Atomic‐resolution STEM image of In_x_Ga_1–x_N/GaN QW lattice showing the ordered atomic arrangements. d) Schematic structure of the on‐chip integrated devices of blue‐LED and PD. e) 2‐inch monolithically integrated device wafer. f) Optical microscopy image of integrated LED and PD devices. g) EL spectra of blue‐LED with a current range from 20 to 350 mA. The inset shows the luminescent photograph of the LED. h) *I–V* curves of PD responding to illumination from LED operated at current of 0 (dark) and 100 mA. i) Induced photocurrent temporal trace of PD at 0 V bias with the cyclical light (100 mA)/dark (0 mA) changes in LED. j) The rise time and decay time of the self‐powered PD under the illumination of LED.

To confirm the feasibility of on‐chip integration of commonly used GaN‐based LED and PD on CMOS‐compatible Si(100), we carry out the growth of multiple quantum wells (MQWs) on as‐grown GaN/Gr/SiO_2_/Si(100) and fabricate the prototype devices. **Figure** **4**a clearly shows five‐period of In_x_Ga_1‐x_N/GaN MQWs with uniform In, Ga, and N elements distribution. The corresponding line profile of In and Ga elements also proves the uniformity of the high‐quality MQWs structure (Figure 4b). In addition, the atomic STEM image of the In_x_Ga_1−x_N/GaN MQWs display regular atomic arrangements along the growth direction [0001] (Figure 4c). For the first time, we fabricate the monolithically integrated device with blue‐LED and PD on the same chip on SiO_2_/Si(100) substrate, as illustrated in Figure 4d,e and Figure S12 (Supporting Information). The InGaN/GaN MQWs, which are responsible for blue light emission in LED, are also used for photodetection in PD, realizing a self‐driven function with zero bias. The area of the active region in diodes is 400 × 120 µm^2^, and the waveguide is 600 µm long as shown in Figure 4f. The normalized EL spectra of as‐fabricated LED under different injection current are shown in Figure 4g. The peak position of LED blueshifts from 462.1 to 453.2 nm as the applied current increases from 20 to 350 mA, due to the residual biaxial stress in MQWs. The inset Figure 4g shows the luminescent photograph of the LED. Utilizing the LED as the light source, the PD can respond to the LED light signal and monitor the fluctuation of LED intensity in real‐time.^[^ ^46^, ^47^ ^]^ As shown in Figure 4h, the PD shows a dark current ≈2.49 × 10^−7^ A at zero bias. Under the blue‐LED radiation at 100 mA, the photocurrent reaches 3.24 × 10^−6^ A with PDCR of 13. The responsivity and specific detectivity are estimated to be 2.01 × 10^5^ A/W and 4.64 × 10^13^ Jones at zero bias, respectively. According to the *I–T* test at zero bias, it can be found that the PD has an obvious optical response, good repeatability, and high stability in Figure 4i. The rise and the decay time are usually measured between 10% and 90% of the maximum photocurrent. Under our test conditions, the rise and the decay time of the PD are 248 µs and 129 µs, respectively (Figure 4j). In comparison with conventional metal‐semiconductor‐metal (MSM) self‐powered GaN PDs,^[^ ^48^ ^]^ it shows two orders of magnitude faster and demonstrates great potential of rapid response.

## 3 Discussion

The smart unification of GaN‐enabled device and Si‐based CMOS holds promise for breaking through the limitation of Moore's law, which is also compatible with conventional Si fabrication technology. As expected, the epitaxy of single‐crystalline GaN film over SiO_2_/Si(100) has been fulfilled. In further contexts, we have demonstrated, for the first time, the monolithic integration of PD and LED on such CMOS‐compatible SiO_2_/Si(100) substrate. These remarkable advancements can be attributed primarily to two critical strategies: the oriented attachment on graphene and multi‐step growth. For one thing, the realization of single‐crystalline GaN film is enabled by oriented attachment strategy, where the in‐plane orientation of AlN on Gr is aligned throughout the slip and rotation mode stemming from the weakened interface interaction. Admittedly, uniform few‐layer Gr, elevated processing temperature and high nuclei density are essential to expedite the oriented attachment during qvdW growth. In this regard, the size of AlN nuclei should be small enough to realize the easy alignment driven by the thermal energy. To this end, the in‐situ NH_3_ annealing treatment can elevate the nucleation density and render a uniform size distribution. For another, the out‐of‐plane orientation of GaN can be guided by hexagonal Gr in a multi‐step growth manner. It is worth‐noting the preferred c‐axis orientation possesses the strongest interfacial interaction with Gr, thereby gradually annex the mis‐orientated nuclei during growth. As a result, the GaN film grown on SiO_2_/Si(100) affords markedly improved quality as compared to those from previous reports. Specifically, the RMS roughness of GaN film is as low as 0.185 nm within a scanned area of 1 × 1 µm^2^ and the FWHM of (0002) peak of GaN film is 0.46°. It is anticipated that the quality of GaN film on SiO_2_/Si(100) could be further improved by optimizing the Gr synthesis, such as improving the uniformity of Gr (the area of bilayer region reaches up to 90%) and reducing the surface‐bound amorphous carbon dots. Equally encouragingly, the monolithic integration of PD and LED on SiO_2_/Si(100) readily enables real‐time light monitoring. At a current density of 100 mA in LED device, the PDCR value of the self‐powered PD reaches up to 13 and the rise and the decay time of the PD are 248 and 129 µs. Meanwhile, the responsivity and detectivity of PD reaches 2.01 × 10^5^ A W^−1^ and 4.64 × 10^13^ Jones, respectively. Collectively, the direct q*v*d*W* strategy not only represents a straightforward route to enhance the system speed and reduce the power consumption, but also offers an attractive opportunity to integrate various 3D and 2D materials into epitaxial heterostructures via circumventing their lattice mismatch.

In summary, large area single‐crystalline GaN film on CMOS‐compatible SiO_2_/Si(100) substrate is successfully realized with the aid of directly grown Gr interlayer. Experimental results and theoretical analysis corroborate that Gr helps elevate the nucleation efficiency of nitrides and further guide their out‐of‐plane and in‐plane crystalline orientation via the oriented attachment and multi‐step growth. Based on the single crystalline GaN/SiO_2_/Si(100) presence‐at‐hand, the first‐time monolithic photonic integration of GaN‐based prototype devices with Si(100) substrate is realized. At zero bias, the PD enables a real‐time and rapid‐response monitoring to the light signal emitted by the LED. This work sheds light on the growth mechanism of high‐quality GaN film over 2D materials and boosts the smart design of nitrides‐based photoelectric devices compatible with Si(100)‐based ICs.

## 4 Experimental Section

### CVD Growth of Gr on SiO_2_/Si(100) Substrate

Typically, 20 pieces of commercial Si(100) substrate were placed on a home‐made substrate holder and then loaded into a high‐temperature furnace. The Gr growth was performed at atmospheric pressure with 800 sccm Ar, 600 sccm H_2_, and 50 sccm CH_4_ at 1050 °C for 5 h.

### MOCVD Growth of GaN on Gr/SiO_2_/Si(100) Substrate

The Gr/SiO_2_/Si(100) substrate was exposed to NH_3_ self‐annealed in the Veeco K300 MOCVD chamber. Trimethylgallium (TMGa), trimethylaluminum (TMAl), and NH_3_ were used as Ga, Al, and N precursors for growing GaN and AlN films; triethylgallium and trimethylindium were used as Ga and In precursors for growing In_x_Ga_1−x_N/GaN layers in the MQWs, respectively. First, the high temperature (HT)‐AlN was grown at a nominal temperature of 1200 °C for 6 min with the NH_3_ flow of 1000 sccm and TMAl flow of 50 sccm, respectively. Then GaN growth procedure: first, the 1st‐GaN layer was grown at 1050 °C for 40 min with the NH_3_ flow of 3000 sccm and TMGa flow of 100 sccm, respectively. Next, the 2nd‐GaN layer was grown at 1050 °C for 40 min with the NH_3_ flow of 6000 sccm and TMGa flow of 100 sccm, respectively. Then, 3rd‐GaN layer was grown at 1080 °C for 60 min with the NH_3_ flow of 8000 sccm and TMGa flow of 100 sccm, respectively. Then five periods of In_x_Ga_1−x_N/GaN MQWs layer were grown at 735 °C/834 °C with 3 nm InGaN well layers and 12 nm GaN barriers. The active layers were capped with a p‐GaN layer deposited at 950 °C with the bis‐cyclopentadienyl magnesium (Cp_2_Mg) flow of 120 sccm, followed by an annealing process at 720 °C for 10 min under N_2_ ambient.

### LED and PD Integrated Device Fabrication

Integrated LED chips were fabricated using a conventional mesa structure. First, the active regions of the LED and the PD are defined by the inductively coupled plasma reactive ion etching (ICP‐RIE, speed: 40 Å s^−1^) with an etching depth of 500 nm. Second, a Ti/Al/Ni/Au (20/60/30/100 nm) multi‐layers are evaporated by an electron beam evaporator (EBE) and lifted off to form the p‐contact metal electrodes of the LED and PD. Then the electrodes are treated by rapid thermal annealing (RTA) at 1000 °C for 30 s in N_2_ ambient to improve the ohmic contact performance. Similarly, a Ni/Au (20/20 nm) multi‐layers are deposited and lifted off to form the n‐contact metal electrodes, followed by RTA at 700 °C for 1 min in N_2_ atmosphere. Subsequently, the structure of the waveguide is defined by photolithography through ICP dry etching. Finally, a high‐quality SiO_2_ passivation protective layer with a thickness of 100 nm is deposited by plasma enhanced chemical vapor deposition (PECVD) and patterned by BOE. Finally, the whole wafer was cut into pieces of devices and then packaged.

### Characterization

The samples were characterized by SEM (Hitachi S‐4800; operating at 3 kV), AFM (D3100, Veeco), Raman spectrum (Horiba, 532 nm laser excitation), XRD (BRUKER D8 Discover High‐resolution XRD system with Cu Kα radiation, *λ* ≈1.5418 Å), XPS (Kratos Analytical Axis‐Ultra spectrometer using a monochromatic Al Kα X‐ray source), EBSD (Thermo Fisher Scientific FEI Quattro S instrument operated at 20 kV), Cross‐sectional TEM (FIB system, Thermo Fisher Helios G4 UX), HRTEM and SAED (FEI Tecnai F20 TEM, operated at 200 kV), HAADF and EDS (FEI Titan Cubed Themis G2 300 spherical aberration‐corrected STEM, operated at 300 kV). In HAADF mode, the camera length was set as 145 mm. The convergence semi‐angle and the collection semi‐angle for HAADF imaging was 30 mrad and 39–200 mrad, respectively. The EL spectrum of blue‐LED was characterized by Integrating sphere (HAAS‐2000). The photoelectronic measurements were performed on an optical platform equipped with an optical system and a semiconductor characterization system (B1500, Keysight).

### Computational Details

The first‐principles calculations based on DFT are performed using the Vienna ab‐initio simulation package (VASP)^[^ ^49^ ^]^ with the generalized‐gradient approximation of Perdew, Burke, and Ernzerhof (PBE)^[^ ^50^ ^]^ for the exchange correlation functional. We use the all‐electron‐like projector‐augmented wave potentials^[^ ^51^ ^]^ and set the energy cutoff for the plane‐wave expansion as 400 eV. Weak van‐der Waals interactions are included with the Becke88 optimization (optB88) functional.^[^ ^52^ ^]^ We adopted the most stable reconstructed SiO_2_(100) surface to model the SiO_2_ substrate. On this reconstructed surface, the top two layers merge together to form three‐membered and six‐membered rings, removing all surface dangling bonds.^[^ ^53^ ^]^ The (100) SiO_2_ reconstructed surface^[^ ^53^ ^]^ is modelled by a six‐unit‐cell thick slab, with the bottom passivated by pseudohydrogen atoms. The bottom two layers of Si and O atoms and the passivated pseudohydrogen atoms are kept fixed during all the structural relaxations. To calculate the adsorption energies of Al atom on different substrates, we introduce one Al atom on (0001) SiO_2_ (2 × 2) supercell or on Gr bilayer (6 × 6) supercell. The N defects are introduced in the first layer of the bilayer Gr. To calculate the sliding energy barriers of AlN on different surfaces, we shift a six‐bilayer thick (0001) AlN film on Gr bilayer and (0001) SiO_2_ reconstructed surface along AlN [10$\bar{1}$0] direction. The top layer of the AlN film is also passivated by pseudohydrogen atoms to eliminate surface effects. To minimize the interface strain, the in‐plane lattice vectors of AlN is rotated by 30° with respect to those of Gr, while they are the same as those of (0001) SiO_2_ surface. To obtain the energy variation during the shifts of the AlN film, we calculate twenty‐one different positions along the shifting path. At each position, the top two layers of the AlN bilayers and the passivated pseudohydrogen atoms are kept fixed, whereas the bottom four AlN bilayers near the interface are fully relaxed. The C atoms in Gr bilayer substrate are allowed to fully relaxed. The rotation of AlN nanorod on Gr is modelled by a small (0001) AlN nanorod with 6.8 Å in diameter and 17.5 Å in length on Gr (6 × 6) supercell.

D.D. Liang, B. Jiang, Z.T. Liu, and Z. L. Chen contributed equally to this work. This work was financially supported by the Beijing Natural Science Foundation (grant 4222077), the National Key R&D Program of China (grants 2019YFA0708203 and 2019YFA0708201), the Beijing Science and Technology Plan (no. Z221100002722019), the National Natural Science Foundation of China (grants 61974139, 52192614, and 62175228) and the CAS Project for Young Scientists in Basic Research (grant YSBR‐064).

## Conflict of Interest

The authors declare no conflict of interest.

## Author Contributions

T.W. and J.S. conceived the idea and designed this work. D.L., Y.G. and L.W. performed the nitride growth experiments and device fabrication under the direction of T.W. and J.W., and Z.T.L. performed the electron microscopy experiments under the direction of P.G. B.J. and Z.C. contributed to the growth of Gr under the direction of J.S. and Z.F.L. and S.Y. carried out the DFT calculations. R.H., J.R. and J.L. performed the XRD and Raman analysis. D.L., B.J., Z.T.L. and Z.C. performed the data analysis and wrote the manuscript under the direction of T.W and J.S. All authors contributed to the discussion and analysis of the results.

## Supporting information

Supporting Information

## Data Availability

The data that support the findings of this study are available from the corresponding author upon reasonable request.
