# Supplementary material for: Quasi van der Waals Epitaxy of Single Crystalline GaN on Amorphous SiO2/Si(100) for Monolithic Optoelectronic Integration
Source: Adv Sci (Weinh). 2024 Mar 22;11(20):2305576. doi: 10.1002/advs.202305576 (PMC11132040; doi:10.1002/advs.202305576)
Supplement: Supplementary file 1 — Supporting Information [file ADVS-11-2305576-s001.pdf]

ADVANCED SCIENCE
Open Access

Supporting Information

for *Adv. Sci.*, DOI 10.1002/advs.202305576

Quasi van der Waals Epitaxy of Single Crystalline GaN on Amorphous SiO_2/Si(100) for Monolithic Optoelectronic Integration

Dongdong Liang, Bei Jiang, Zhetong Liu, Zhaolong Chen, Yaqi Gao, Shenyuan Yang, Rui He, Lulu Wang, Junxue Ran, Junxi Wang, Peng Gao, Jinmin Li, Zhongfan Liu, Jingyu Sun* and Tongbo Wei**

Supplementary Information

Quasi van der Waals epitaxy of single crystalline GaN on amorphous SiO_2/Si(100) for monolithic optoelectronic integration

Dongdong Liang, Bei Jiang, Zhetong Liu, Zhaolong Chen, Yaqi Gao, Shenyuan Yang*, Rui He, Lulu Wang, Junxue Ran, Junxi Wang, Peng Gao, Jinmin Li, Zhongfan Liu, Jingyu Sun*, Tongbo Wei*

D.D. Liang, Y.Q. Gao, R. He, L.L. Wang, J.X. Ran, Prof. J.X. Wang, Prof. J.M. Li, Prof. T.B. Wei
Research and Development Center for Semiconductor Lighting Technology, Institute of Semiconductors, Chinese Academy of Sciences, Beijing, 100083, China
E-mail: tbwei@semi.ac.cn

D.D. Liang, Y.Q. Gao, Prof. S.Y. Yang, R. He, L.L. Wang, J.X. Ran, Prof. J.X. Wang, Prof. J.M. Li, Prof. T.B. Wei
Center of Materials Science and Optoelectronics Engineering, University of Chinese Academy of Sciences, Beijing 100049, China
E-mail: syyang@semi.ac.cn

B. Jiang, Z.T. Liu, Z.L. Chen, Prof. P. Gao, Prof. Z.F. Liu
Center for Nanochemistry (CNC), Beijing Science and Engineering Center for Nanocarbons, Beijing National Laboratory for Molecular Sciences, College of Chemistry and Molecular Engineering, Peking University, Beijing 100871, China

Z.T. Liu, Prof. P. Gao
Electron Microscopy Laboratory, and International Center for Quantum Materials, School of Physics, Peking University, Beijing, 100871, China

Prof. S.Y. Yang

State Key Laboratory of Superlattices and Microstructures, Institute of Semiconductors, Chinese Academy of Sciences, Beijing 100083, China

B. Jiang, Z.T. Liu, Z.L. Chen, Prof. P. Gao, Prof. Z.F. Liu, Prof. J.Y. Sun
Beijing graphene institute (BGI), Beijing, 100095, P. R. China

Prof. Z.F. Liu, Prof. J.Y. Sun
College of Energy, Soochow Institute for Energy and Materials InnovationS (SIEMIS), Jiangsu Provincial Key Laboratory for Advanced Carbon Materials and Wearable Energy Technologies, Soochow University, 215006, Suzhou, P. R. China
E-mail: sunjy86@suda.edu.cn

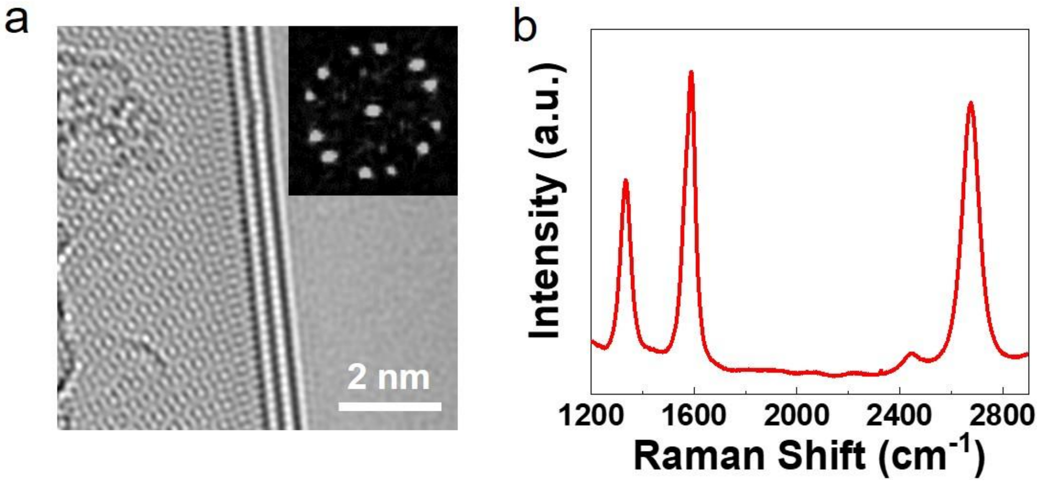

Figure S1. a) HRTEM image of directly grown Gr. The inset shows the corresponding FFT pattern. **b)** The typical Raman spectrum of directly grown Gr, displaying the featured G peak at 1590 cm^{-1} and 2D peak at 2685 cm^{-1}.

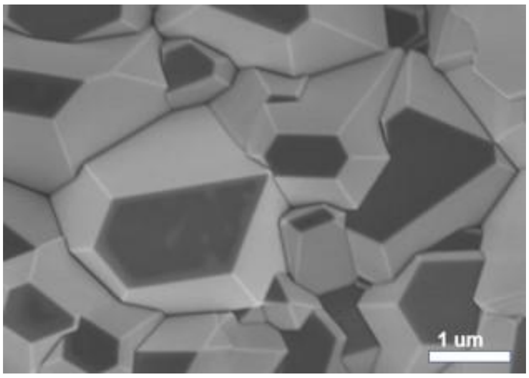

Figure S2. SEM characterization of polycrystalline GaN film grown on SiO_2/Si(100) without Gr interlayer.

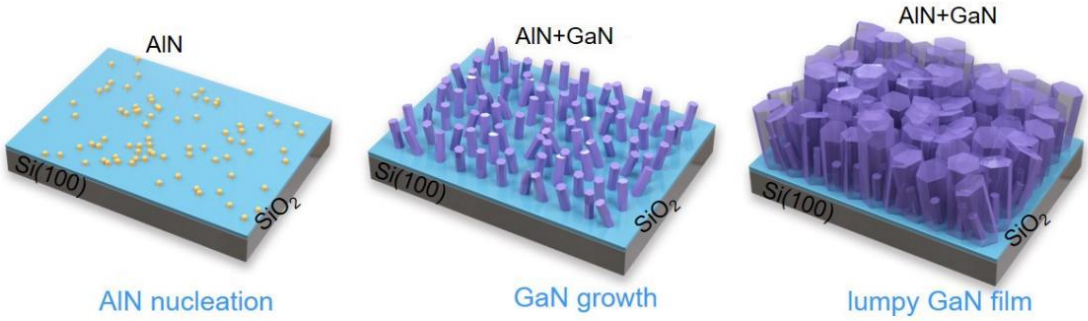

Figure S3. Schematic diagram of the GaN growth model without Gr.

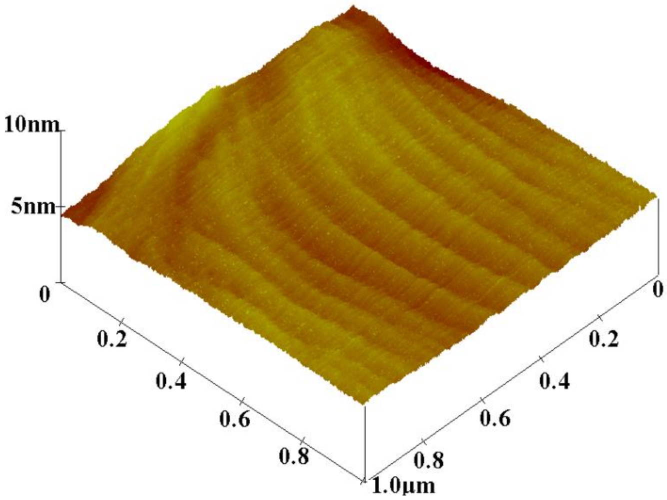

Figure S4. AFM image of top surface morphology for GaN grown on Gr/SiO_2/Si(100) substrate.

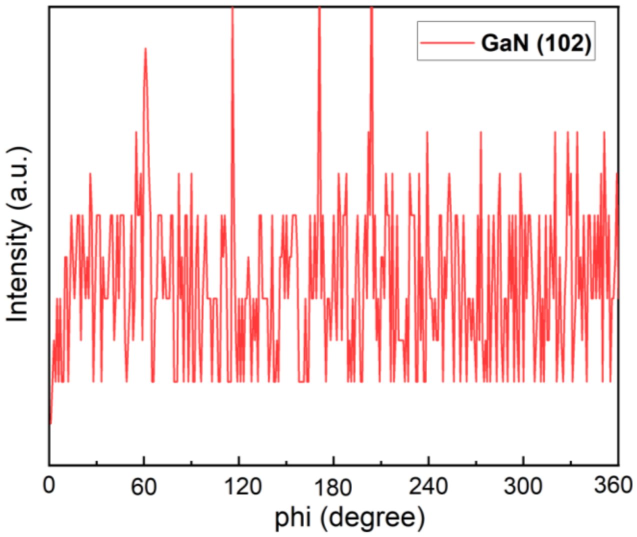

Figure S5. XRD ϕ-scan of GaN ($10\bar{1}2$) grown on bare SiO_2/Si(100) substrate.

Table S1. Summary of the recent progress of GaN epilayers grown on polycrystalline substrates.

Substrate	Epitaxial equipment	Epitaxial layer	FWHM (0002) (°)	AFM (nm)	Device	Year	Ref.
Gr/SiO_2	MOCVD	GaN film/ZnO nanowires	0.8	11 (5*5 μm^2)	LED	2012	[1]
Gr/SiO_2/Si(100)	MOCVD	GaN micro-rod	2.5	--	LED & Flexible LED	2014	[2]
Gr/SiO_2/Si(100)	PA-MBE	GaN nanowires	1.62	--	No	2016	[3]
Gr/SiO_2/Si(100)	MOCVD	GaN film	--	0.46 (3.2*3.2 μm^2)	No	2019	[4]
Single-crystalline Gr/PVD AlN	MOCVD	GaN film	0.59	0.35 (1.8*1.8 μm^2)	No	2022	[5]
Multilayer Gr/SiO_2	Pulsed Sputtering Deposition	GaN film	0.617	--	No	2014	[6]
Gr/Glass	MOCVD	GaN film	1.2	--	LED & Flexible LED	2021	[7]

Gr/Glass	MOCVD	GaN film	1.19	0.24 (1*1 μm^2)	PD	2022	[8]
Gr/Glass	MOCVD	Polycrystal -line GaN film	Polycry- stalline	2.92 (2*2 μm^2)	No	2023	[9]
WS_2-glass	MOCVD	GaN film	1.6	1.898 (2*2 μm^2)	LED	2022	[10]
WS_2-glass	MOCVD	GaN film	1.5	1.83 (4.2*4.2 μm^2)	No	2023	[9]
BN/Glass	MOCVD	GaN film	2.37	--	No	2017	[11]
SiO_2/Si(100)	MOCVD	GaN film	0.46	0.185 (1*1 μm^2)	LED &PD	2023	This work

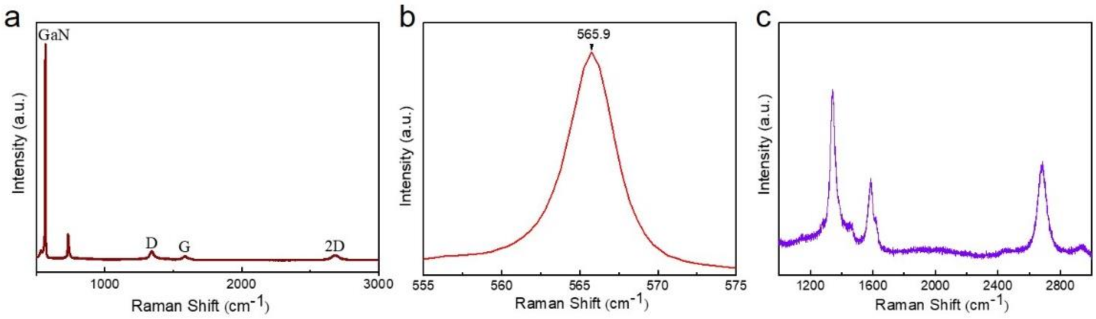

Figure S6. a) Raman spectra of GaN/AlN/Gr/SiO_2/Si(100) with a thickness of 4 μm. **b)** Raman spectra corresponding to E_2 mode of GaN film in **a**. **c)** Raman spectra of Gr corresponding in **a.** The narrow FWHM of GaN E_2 peak also proves the good quality of GaN grown on Gr/SiO_2/Si(100).

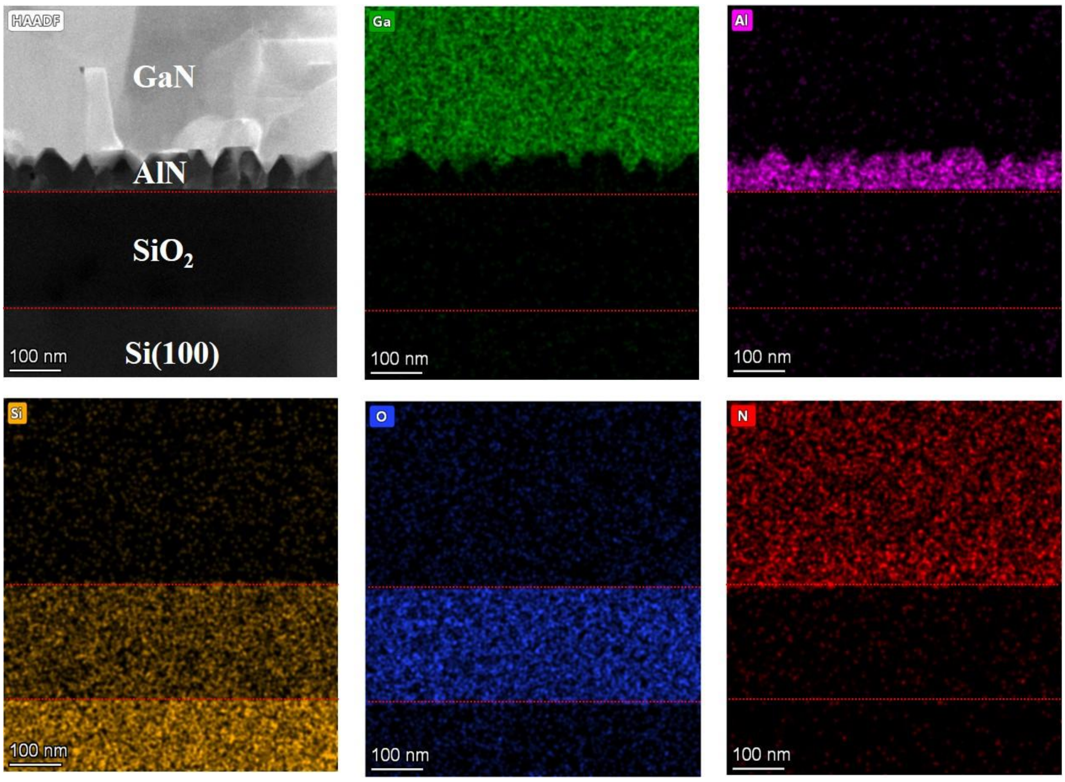

Figure S7. Cross-sectional HR-STEM images taken at the interface region, giving the sharp interface structure of the sample. The EDS mapping of the K-edge of Ga, In, Al, Si and O at the interface region, respectively. The low magnification cross-sectional STEM image of the GaN/AlN/Gr/SiO_2/Si interface and corresponding EDS mapping images of Ga, Al, Si, O and N elements are presented in Figure S6. These images provide a clear illustration of the sample's structure, particularly highlighting the facile coalescence of AlN nuclear islands due to their uniform lattice orientation.

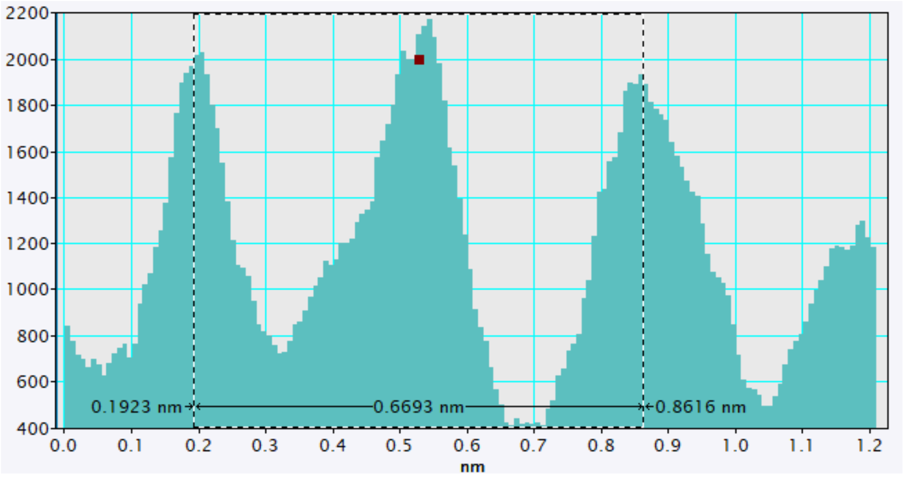

Figure S8. The typical distance of the Gr interlayer is around 0.3346 nm. As depicted in Figure S8, the interlayer distance is approximately 0.3346 nm, which is consistent with that of graphene. Moreover, graphene is still evident after the epitaxy process as evidenced by the line profile, which demonstrates the involvement of graphene in the epitaxial growth process.

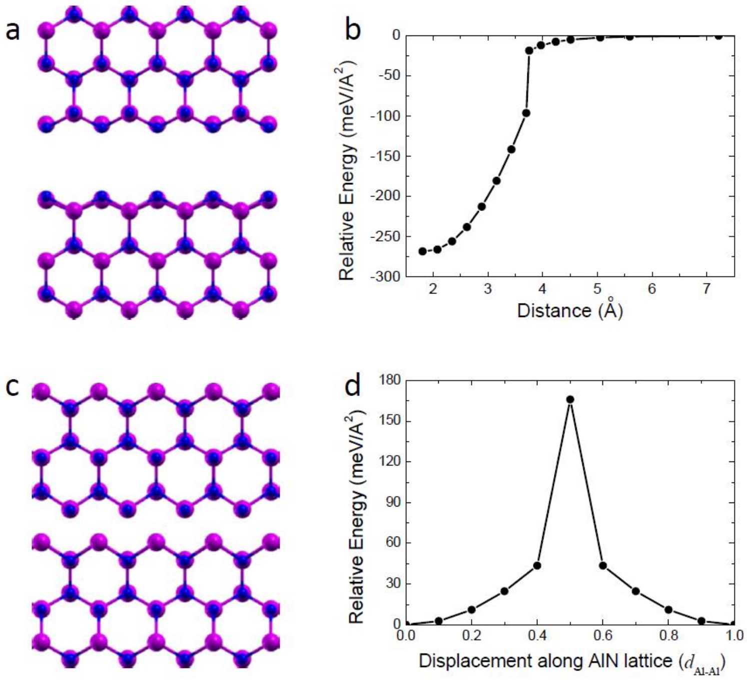

Figure S9. Two grain boundaries of AlN $(10\bar{1}0)$ facets. **a** The grain boundary is formed by increasing the interlayer distance between two AlN surfaces. **b** The energy variation of boundary in **a** as a function of interlayer distance. **c** The grain boundary is formed

by laterally displacing one AlN surface with respect to another. **d** The energy variation of boundary in **c** as a function of displacement.

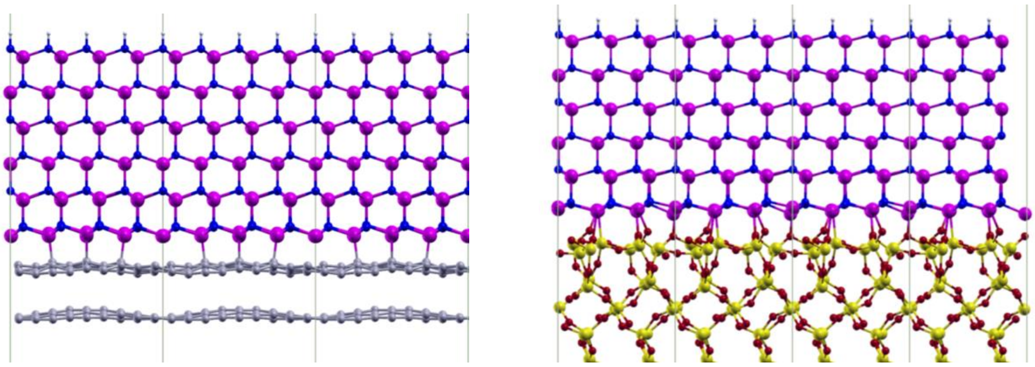

Figure S10. Prototypes of interface structures of AlN (0001) film sliding on Gr bilayer (left) and SiO_2 (0001) reconstructed surfaces (right). The gray vertical lines indicate the size of the supercell. Al atoms can form strong covalent bonds with the surface O atoms on SiO_2 surface. The sliding process involves the bond breaking and bond making at the interface, resulting in a large energy barrier. On Gr surface, the Al-C bonds are weaker and fewer, and the corresponding energy barrier is much smaller.

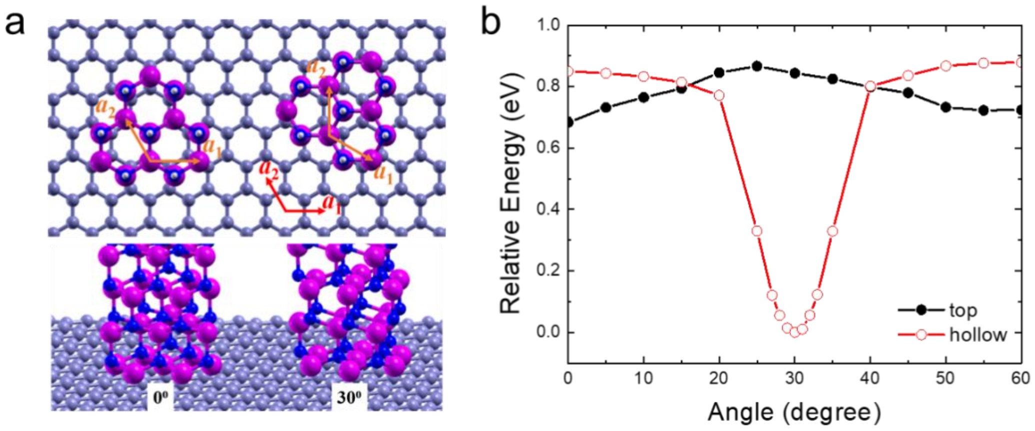

Figure S11. a) Two stable adsorption configurations of AlN nanorod on Gr. The center of the nanorod can be adsorbed on top and hollow sites, with the most stable rotational angles of 0° and 30°, respectively. The red and orange arrows denote the in-plane lattice vectors of Gr and AlN, respectively. **b)** Relative energy of AlN nanorod on Gr as a function of different in-plane rotational angles in the range of 0°-60°. The maximum rotation energy barrier of 0.81 eV corresponds to the rotation of AlN nanorod from 30°

to 0° on hollow site. The cross-section area is estimated from bulk AlN, and the energy barrier per area is calculated to be 16.0 meV/Å^2.

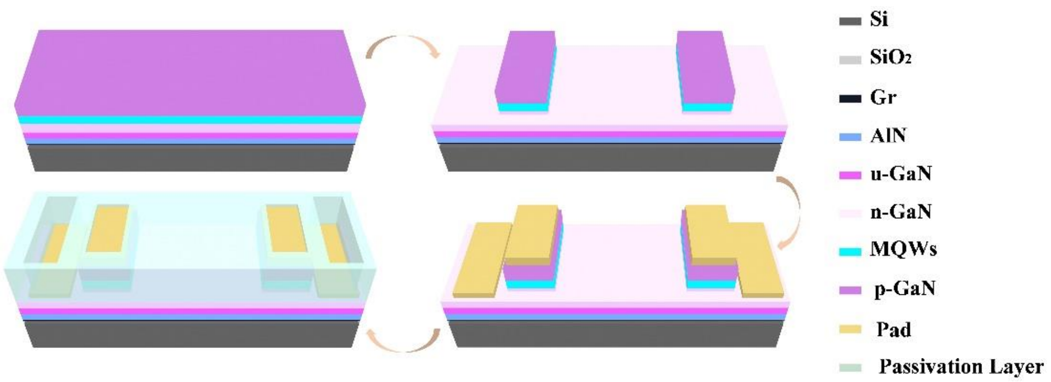

Figure S12. Fabrication processes of the monolithically integrated devices.

REFERENCES

[1] Chung K, In Park S, Baek H, Chung J-S, Yi G-C, High-Quality GaN Films Grown on Chemical Vapor-Deposited Graphene Films. *NPG Asia Mater.* **2012**, 4, e24-e24.

[2] Chung K, Beak H, Tchoe Y, Oh H, Yoo H, Kim M, Yi G-C, Growth and Characterizations of GaN Micro-Rods on Graphene Films for Flexible Light Emitting Diodes. *APL Mater.* **2014**, 2, 092512.

[3] Kumaresan V, Largeau L, Madouri A, Glas F, Zhang H, Oehler F, Cavanna A, Babichev A, Travers L, Gogneau N, Tchernycheva M, Harmand J C, Epitaxy of GaN Nanowires on Graphene. *Nano Lett.* **2016**, 16, 4895-902.

[4] Feng Y, Yang X, Zhang Z, Kang D, Zhang J, Liu K, Li X, Shen J, Liu F, Wang T, Ji P, Xu F, Tang N, Yu T, Wang X, Yu D, Ge W, Shen B, Epitaxy of Single-Crystalline GaN Film on CMOS-Compatible Si(100) Substrate Buffered by Graphene. *Adv. Funct. Mater.* **2019**, 29, 1905056.

[5] Liu D, Hu L, Yang X, Zhang Z, Yu H, Zheng F, Feng Y, Wei J, Cai Z, Chen Z, Ma C, Xu F, Wang X, Ge W, Liu K, Huang B, Shen B, Polarization-Driven-Orientation Selective Growth of Single-Crystalline III-Nitride Semiconductors on Arbitrary Substrates. *Adv. Funct. Mater.* **2022**, 32. 2113211.

[6] Shon J W, Ohta J, Ueno K, Kobayashi A, Fujioka H, Structural Properties of GaN Films Grown on Multilayer Graphene Films by Pulsed Sputtering. *Appl. Phys. Express*. **2014**, 7, 085502.

[7] Ren F, Liu B, Chen Z, Yin Y, Sun J, Zhang S, Jiang B, Liu B, Liu Z, Wang J, Liang M, Yuan G, Yan J, Wei T, Yi X, Wang J, Zhang Y, Li J, Gao P, Liu Z, Liu Z, Van Der Waals Epitaxy of Nearly Single-Crystalline Nitride Films on Amorphous Graphene-Glass Wafer. *Sci. Adv.* **2021**, 7, eabf5011.

[8] Jiang B, Liang D, Sun Z, Ci H, Liu B, Gao Y, Shan J, Yang X, Rümmeli M H, Wang J, Wei T, Sun J, Liu Z, Toward Direct Growth of Ultra-Flat Graphene. Adv. Funct. Mater. **2022**, 32, 2200428.

[9] Chen Q, Yang K, Shi B, Yi X, Wang J, Li J, Liu Z, Principles for 2D-Material-Assisted Nitrides Epitaxial Growth. *Adv. Mater.* 2023, 35, e2211075.

[10] Yin Y, Liu B, Chen Q, Chen Z, Ren F, Zhang S, Liu Z, Wang R, Liang M, Yan J, Sun J, Yi X, Wei T, Wang J, Li J, Liu Z, Gao P, Liu Z, Continuous Single-Crystalline GaN Film Grown on WS_2-Glass Wafer. *Small* **2022**, 18, e2202529.

[11] Chung K, Oh H, Jo J, Lee K, Kim M, Yi G-C, Transferable Single-Crystal GaN Thin Films Grown on Chemical Vapor-Deposited Hexagonal BN Sheets. *NPG Asia Mater.* **2017**, 9, e410-e410.
